# New construction and proof techniques of projection algorithm for countable maximal monotone mappings and weakly relatively non-expansive mappings in a Banach space

**DOI:** 10.1186/s13660-018-1657-3

**Published:** 2018-03-27

**Authors:** Li Wei, Ravi P. Agarwal

**Affiliations:** 1 School of Mathematics and Statistics, Hebei University of Economics and Business, Shijiazhuang, China; 2 Department of Mathematics, Texas A&M University-Kingsville, Kingsville, USA

**Keywords:** 47H05, 47H09, 47H10, Maximal monotone mapping, Weakly relatively non-expansive mapping, Projection, Limit of a sequence of sets, Uniformly convex and uniformly smooth Banach space

## Abstract

In a real uniformly convex and uniformly smooth Banach space, some new monotone projection iterative algorithms for countable maximal monotone mappings and countable weakly relatively non-expansive mappings are presented. Under mild assumptions, some strong convergence theorems are obtained. Compared to corresponding previous work, a new projection set involves projection instead of generalized projection, which needs calculating a Lyapunov functional. This may reduce the computational labor theoretically. Meanwhile, a new technique for finding the limit of the iterative sequence is employed by examining the relationship between the monotone projection sets and their projections. To check the effectiveness of the new iterative algorithms, a specific iterative formula for a special example is proved and its computational experiment is conducted by codes of Visual Basic Six. Finally, the application of the new algorithms to a minimization problem is exemplified.

## Introduction and preliminaries

Let *E* be a real Banach space with $E^{*}$ its dual space. Suppose that *C* is a nonempty closed and convex subset of *E*. The symbol 〈⋅, ⋅〉 denotes the generalized duality pairing between *E* and $E^{*}$. The symbols “→” and “⇀” denote strong and weak convergence either in *E* or in $E^{*}$, respectively.

A Banach space *E* is said to be strictly convex [1] if for $\forall x,y \in E$ which are linearly independent,

$$ \Vert x + y \Vert < \Vert x \Vert + \Vert y \Vert . $$

The above inequality is equivalent to the following:

$$ \Vert x \Vert = \Vert y \Vert = 1, \quad x \ne y \quad \Rightarrow \quad \biggl\Vert \frac{x + y}{2} \biggr\Vert < 1. $$

A Banach space *E* is said to be uniformly convex [1] if for any two sequences $\{ x_{n} \} $ and $\{ y_{n} \} $ in *E* such that $\Vert x_{n} \Vert = \Vert y_{n} \Vert = 1$ and $\lim_{n \to \infty} \Vert x_{n} + y_{n} \Vert = 2$, $\lim _{n \to \infty } \Vert x_{n} - y_{n} \Vert = 0$ holds.

If *E* is uniformly convex, then it is strictly convex.

The function $\rho_{E}:[0, + \infty ) \to [0, + \infty )$ is called the modulus of smoothness of *E* [2] if it is defined as follows:

$$ \rho_{E}(t) = \sup \biggl\{ \frac{1}{2} \bigl(\Vert x + y \Vert + \Vert x - y \Vert \bigr) - 1:x,y \in E, \Vert x \Vert = 1, \Vert y \Vert \le t \biggr\} . $$

A Banach space *E* is said to be uniformly smooth [2] if $\frac{\rho_{E}(t)}{t} \to 0$, as $t \to 0$.

The Banach space *E* is uniformly smooth if and only if $E^{*}$ is uniformly convex [2].

We say *E* has Property (H) if for every sequence $\{ x_{n}\} \subset E$ which converges weakly to $x \in E$ and satisfies $\Vert x_{n} \Vert \to \Vert x \Vert $ as $n \to \infty $ necessarily converges to *x* in the norm.

If *E* is uniformly convex and uniformly smooth, then *E* has Property (H).

With each $x \in E$, we associate the set

$$ J(x) = \bigl\{ f \in E^{*}: \langle x,f \rangle = \Vert x \Vert ^{2} = \Vert f \Vert ^{2} \bigr\} ,\quad \forall x \in E. $$

Then the multi-valued mapping $J:E \to 2^{E^{*}}$ is called the normalized duality mapping [1]. Now, we list some elementary properties of *J*.

### Lemma 1.1

([1, 2])

1. *If* *E* *is a real reflexive and smooth Banach space*, *then* *J* *is single valued*;
2. *if* *E* *is reflexive*, *then* *J* *is surjective*;
3. *if* *E* *is uniformly smooth and uniformly convex*, *then* $J^{ - 1}$ *is also the normalized duality mapping from* $E^{*}$ *into E*. *Moreover*, *both* *J* *and* $J^{ - 1}$ *are uniformly continuous on each bounded subset of* *E* *or* $E^{*}$, *respectively*;
4. *for* $x \in E$ *and* $k \in ( - \infty, + \infty )$, $J(kx) = kJ(x)$.

For a nonlinear mapping *U*, we use $F(U)$ and $N(U)$ to denote its fixed point set and null point set, respectively; that is, $F(U) = \{ x \in D(U):Ux = x\}$ and $N(U) = \{ x \in D(U):Ux = 0\}$.

### Definition 1.2

([3])

A mapping $T \subset E \times E^{*}$ is said to be monotone if, for $\forall y_{i} \in Tx_{i}$, $i = 1,2$, we have $\langle x_{1} - x_{2},y_{1} - y_{2} \rangle \ge 0$. The monotone mapping *T* is called maximal monotone if $R(J + \theta T) = E^{*}$ for $\theta > 0$.

### Definition 1.3

([4])

The Lyapunov functional $\varphi :E \times E^{*} \to (0, + \infty )$ is defined as follows:

$$ \varphi (x,y) = \Vert x \Vert ^{2} - 2 \bigl\langle x,j(y) \bigr\rangle + \Vert y \Vert ^{2},\quad \forall x,y \in E,j(y) \in J(y). $$

### Definition 1.4

([5])

Let $B:C \to C$ be a mapping, then

1. an element $p \in C$ is said to be an asymptotic fixed point of *B* if there exists a sequence $\{ x_{n}\}$ in *C* which converges weakly to *p* such that $x_{n} - Bx_{n} \to 0$, as $n \to \infty $. The set of asymptotic fixed points of *B* is denoted by $\hat{F}(B)$;
2. $B:C \to C$ is said to be strongly relatively non-expansive if $\hat{F}(B) = F(B) \ne \emptyset $ and $\varphi (p,Bx) \le \varphi (p,x)$ for $x \in C$ and $p \in F(B)$;
3. an element $p \in C$ is said to be a strong asymptotic fixed point of *B* if there exists a sequence $\{ x_{n}\}$ in *C* which converges strongly to p such that $x_{n} - Bx_{n} \to 0$, as $n \to \infty $. The set of strong asymptotic fixed points of *B* is denoted by $\tilde{F}(B)$;
4. $B:C \to C$ is said to be weakly relatively non-expansive if $\tilde{F}(B) = F(B) \ne \emptyset $ and $\varphi (p,Bx) \le \varphi (p,x)$ for $x \in C$ and $p \in F(B)$.

### Remark 1.5

It is easy to see that strongly relatively non-expansive mappings are weakly relatively non-expansive mappings. However, an example in [6] shows that a weakly relatively non-expansive mapping is not a strongly relatively non-expansive mapping.

### Lemma 1.6

([5])

*Let* *E* *be a uniformly convex and uniformly smooth Banach space and* *C* *be a nonempty closed and convex subset of E*. *If* $B:C \to C$ *is weakly relatively non*-*expansive*, *then* $F(B)$ *is a closed and convex subset of E*.

### Lemma 1.7

([3])

*Let* $T \subset E \times E^{*}$ *be maximal monotone*, *then*

1. $N(T)$ *is a closed and convex subset of* *E*;
2. *if* $x_{n} \to x$ *and* $y_{n} \in Tx_{n}$ *with* $y_{n} \rightharpoonup y$, *or* $x_{n} \rightharpoonup x$ *and* $y_{n} \in Tx_{n}$ *with* $y_{n} \to y$, *then* $x \in D(T)$ *and* $y \in Tx$.

### Definition 1.8

([4])

1. If *E* is a reflexive and strictly convex Banach space and *C* is a nonempty closed and convex subset of *E*, then for each $x \in E$ there exists a unique element $v \in C$ such that $\Vert x - v \Vert = \inf \{ \Vert x - y \Vert :y \in C\}$. Such an element *v* is denoted by $P_{C}x$ and $P_{C}$ is called the metric projection of *E* onto *C*.
2. Let *E* be a real reflexive, strictly convex, and smooth Banach space and *C* be a nonempty closed and convex subset of *E*, then for $\forall x \in E$, there exists a unique element $x_{0} \in C$ satisfying $\varphi (x_{0},x) = \inf \{ \varphi (y, x) :y \in C\}$. In this case, $\forall x \in E$, define $\Pi_{C}:E \to C$ by $\Pi_{C}x = x_{0}$, and then $\Pi_{C}$ is called the generalized projection from *E* onto *C*.

It is easy to see that $\Pi_{C}$ is coincident with $P_{C}$ in a Hilbert space.

Maximal monotone mappings and weakly or strongly relatively non-expansive mappings are different types of important nonlinear mappings due to their practical background. Much work has been done in designing iterative algorithms either to approximate a null point of maximal monotone mappings or a fixed point of weakly or strongly relatively non-expansive mappings, see [5–10] and the references therein. It is a natural idea to construct iterative algorithms to approximate common solutions of a null point of maximal monotone mappings and a fixed point of weakly or strongly relatively non-expansive mappings, which can be seen in [11–15] and the references therein. Now, we list some closely related work.

In [12], Wei et al. presented the following iterative algorithms to approximate a common element of the set of null points of the maximal monotone mapping $T \subset E \times E^{*}$ and the set of fixed points of the strongly relatively non-expansive mapping $S \subset E \times E$, where *E* is a real uniformly convex and uniformly smooth Banach space:

1.1 $$\begin{aligned}& \textstyle\begin{cases} x_{1} \in E,\quad r_{1} > 0, \\ y_{n} = (J + r_{n}T)^{ - 1}J(x_{n} + e_{n}), \\ z_{n} = J^{ - 1}[\alpha_{n}Jx_{n} + (1 - \alpha_{n})Jy_{n}], \\ u_{n} = J^{ - 1}[\beta_{n}Jx_{n} + (1 - \beta_{n})JSz_{n}], \\ H_{n} = \{ z \in E:\varphi (z,z_{n}) \le \alpha_{n}\varphi (z,x_{n}) + (1 - \alpha_{n})\varphi (z,x_{n} + e_{n})\}, \\ V_{n} = \{ z \in E:\varphi (z,u_{n}) \le \beta_{n}\varphi (z,x_{n}) + (1 - \beta_{n})\varphi (z,z_{n})\}, \\ W_{n} = \{ z \in E: \langle z - x_{n},Jx_{1} - Jx _{n} \rangle \le 0\}, \\ x_{n + 1} = \Pi_{H_{n} \cap V_{n} \cap W_{n}}(x_{1}),\quad n \in N, \end{cases}\displaystyle \end{aligned}$$

1.2 $$\begin{aligned}& \textstyle\begin{cases} x_{1} \in E,\quad r_{1} > 0, \\ y_{n} = (J + r_{n}T)^{ - 1}J(x_{n} + e_{n}), \\ z_{n} = J^{ - 1}[\alpha_{n}Jx_{1} + (1 - \alpha_{n})Jy_{n}], \\ u_{n} = J^{ - 1}[\beta_{n}Jx_{1} + (1 - \beta_{n})JSz_{n}], \\ H_{n} = \{ z \in E:\varphi (z,z_{n}) \le \alpha_{n}\varphi (z,x_{1}) + (1 - \alpha_{n})\varphi (z,x_{n} + e_{n})\}, \\ V_{n} = \{ z \in E:\varphi (z,u_{n}) \le \beta_{n}\varphi (z,x_{1}) + (1 - \beta_{n})\varphi (z,z_{n})\}, \\ W_{n} = \{ z \in E:\langle z - x_{n},Jx_{1} - Jx _{n}\rangle \le 0\}, \\ x_{n + 1} = \Pi_{H_{n} \cap V_{n} \cap W_{n}}(x_{1}),\quad n \in N, \end{cases}\displaystyle \end{aligned}$$

and

1.3 $$ \textstyle\begin{cases} x_{1} \in E,\quad r_{1} > 0, \\ y_{n} = (J + r_{n}T)^{ - 1}J(x_{{n}} + e_{n}), \\ z_{n} = J^{ - 1}[\alpha_{n}Jx_{n} + (1 - \alpha_{n})Jy_{n}], \\ u_{n} = J^{ - 1}[\beta_{n}Jx_{n} + (1 - \beta_{n})JSz_{n}], \\ H_{1} = \{ z \in E:\varphi (z,z_{1}) \le \alpha_{1}\varphi (z,x_{1}) + (1 -\alpha_{1})\varphi (z,x_{1} + e_{1})\}, \\ V_{1} = \{ z \in E:\varphi (z,u_{1}) \le \beta_{1}\varphi (z,x_{1}) + (1 - \beta_{1})\varphi (z,z_{1})\}, \\ W_{1} = E, \\ H_{n} = \{ z \in H_{n - 1} \cap V_{n - 1} \cap W_{n - 1}:\varphi (z,z _{n}) \le \alpha_{n}\varphi (z,x_{n}) + (1 - \alpha_{n})\varphi (z,x_{n} + e _{n})\}, \\ V_{n} = \{ z \in H_{n - 1} \cap V_{n - 1} \cap W_{n - 1}:\varphi (z,u _{n}) \le \beta_{n}\varphi (z,x_{n}) + (1 - \beta_{n})\varphi (z,z_{n})\}, \\ W_{n} = \{ z \in H_{n - 1} \cap V_{n - 1} \cap W_{n - 1}: \langle z - x_{n},Jx_{1} - Jx_{n} \rangle \le 0\}, \\ x_{n + 1} = \Pi_{H_{n} \cap V_{n} \cap W_{n}}(x_{1}),\quad n \in N. \end{cases} $$

Under some mild assumptions, $\{ x_{n}\}$ generated by (1.1), (1.2), or (1.3) is proved to be strongly convergent to $\Pi_{N(T) \cap F(S)}(x _{1})$. Compared to projective iterative algorithms (1.1) and (1.2), iterative algorithm (1.3) is called monotone projection method since the projection sets $H_{{n}}$, $V_{n}$, and $W_{n}$ are all monotone in the sense that $H_{n + 1} \subset H_{n}$, $V_{n + 1} \subset V_{n}$, and $W_{{n} + 1} \subset W_{n}$ for $n \in N$. Theoretically, the monotone projection method will reduce the computation task.

In [13], Klin-eam et al. presented the following iterative algorithm to approximate a common element of the set of null points of the maximal monotone mapping $A \subset E \times E^{*}$ and the sets of fixed points of two strongly relatively non-expansive mappings $S,T \subset C \times C$, where *C* is the nonempty closed and convex subset of a real uniformly convex and uniformly smooth Banach space *E*.

1.4 $$ \textstyle\begin{cases} u_{n} = J^{ - 1}[\alpha_{n}Jx_{n} + (1 - \alpha_{n})JT{z}_{ {n}}], \\ {z}_{n} = J^{ - 1}[\beta_{n}Jx_{n} + (1 - \beta_{n})JS( {J} + {r}_{{n}}{A})^{ - 1}{Jx}_{ {n}}], \\ H_{n} = \{ z \in C:\varphi (z,u_{n}) \le \varphi (z,x_{n})\}, \\ V_{n} = \{ z \in C: \langle z - x_{n},Jx_{1} - Jx_{n} \rangle \le 0\}, \\ x_{n + 1} = \Pi_{H_{n} \cap V_{n}}(x_{1}),\quad n \in N. \end{cases} $$

Under some assumptions, $\{ x_{n}\}$ generated by (1.4) is proved to be strongly convergent to $\Pi_{N(A) \cap F(S) \cap F(T)}(x_{1})$.

In [14], Wei et al. extended the topic to the case of finite maximal monotone mappings $\{ T_{i}\}_{i = 1}^{{m}_{1}}$ and finite strongly relatively non-expansive mappings $\{ S_{j}\}_{j = 1}^{ {m}_{2}}$. They constructed the following two iterative algorithms in a real uniformly convex and uniformly smooth Banach space *E*:

1.5 $$ \textstyle\begin{cases} x_{1} \in E,\quad r > 0, \\ y_{n} = J^{ - 1}[\beta_{{n}}Jx_{n} + \sum_{i = 1}^{m_{1}} \beta_{n,i}J(J + rT_{i})^{ - 1}Jx_{n}], \\ x_{n + 1} = J^{ - 1}[\alpha_{n}Jx_{n} + \sum_{j = 1}^{m_{2}}\alpha_{n,j}J S_{j} y_{n}],\quad n \in N, \end{cases} $$

and

1.6 $$ \textstyle\begin{cases} x_{1} \in E,\quad r > 0, \\ y_{n} = J^{ - 1}[\beta_{{n}}Jx_{n} + (1 - \beta_{n})J(J + rT _{1})^{ - 1}J(J + rT_{2})^{ - 1}J \cdots (J + rT_{m_{1}})^{ - 1}Jx_{n}], \\ x_{n + 1} = J^{ - 1}[\alpha_{n}Jx_{n} + (1 - \alpha_{n})JS_{1}S_{2}\cdots S_{m_{2}}y_{n}],\quad n \in N. \end{cases} $$

Under some assumptions, $\{ x_{n}\}$ generated by (1.5) or (1.6) is proved to be weakly convergent to $v = \lim_{n \to \infty } \Pi_{( \bigcap_{i = 1}^{m_{1}}N(T_{i})) \cap ( \bigcap_{j = 1}^{m_{2}}F(S _{j}))}(x_{{n}})$.

Inspired by the previous work, in Sect. 2.1, we shall construct some new iterative algorithms to approximate the common element of the sets of null points of countable maximal monotone mappings and the sets of fixed points of countable weakly relatively non-expansive mappings. New proof techniques can be found, restrictions are mild, and error is considered. In Sect. 2.2, an example is listed and a specific iterative formula is proved. Computational experiments which show the effectiveness of the new abstract iterative algorithms are conducted. In Sect. 2.3, an application to the minimization problem is demonstrated.

The following preliminaries are also needed in our paper.

### Definition 1.9

([16])

Let $\{ C_{n}\}$ be a sequence of nonempty closed and convex subsets of *E*, then

1. $s\mbox{-}\lim \inf C_{n}$, which is called strong lower limit, is defined as the set of all $x \in E$ such that there exists $x_{n} \in C_{n}$ for almost all *n* and it tends to *x* as $n \to \infty $ in the norm.
2. $w\mbox{-}\lim \sup C_{n}$, which is called weak upper limit, is defined as the set of all $x \in E$ such that there exists a subsequence $\{ C_{n_{k}}\}$ of $\{ C_{n}\}$ and $x_{n_{k}} \in C_{n_{k}}$ for every $n_{k}$ and it tends to *x* as $n_{k} \to \infty $ in the weak topology;
3. if $s\mbox{-}\lim \inf C_{n} = w\mbox{-}\lim \sup C_{n}$, then the common value is denoted by $\lim C_{n}$.

### Lemma 1.10

([16])

*Let* $\{ C_{n}\}$ *be a decreasing sequence of closed and convex subsets of* *E*, *i*.*e*., $C_{n} \subset C_{m}$ *if* $n \ge m$. *Then* $\{ C_{n}\}$ *converges in E and* $\lim C_{n} = \bigcap_{n = 1}^{\infty } C_{n}$.

### Lemma 1.11

([17])

*Suppose that* *E* *is a real reflexive and strictly convex Banach space*. *If* $\lim C_{n}$ *exists and is not empty*, *then* $\{ P_{c_{n}}x\}$ *converges weakly to* $P_{\lim C_{n}}x$ *for every* $x \in E$. *Moreover*, *if* *E* *has Property* (H), *the convergence is in norm*.

### Lemma 1.12

([18])

*Let* *E* *be a real smooth and uniformly convex Banach space*, *and let* $\{ u_{n}\}$ *and* $\{ v_{n}\}$ *be two sequences of E*. *If either* $\{ u_{n}\}$ *or* $\{ v_{n}\}$ *is bounded and* $\varphi (u_{n},v_{n}) \to 0$, *as* $n \to \infty $, *then* $u_{n} - v_{n} \to 0$, *as* $n \to \infty $.

### Lemma 1.13

([19])

*Let* *E* *be a real uniformly convex Banach space and* $r \in (0, + \infty )$. *Then there exists a continuous*, *strictly increasing*, *and convex function* $\omega : [0,2r] \to [0, +\infty)$ *with* $\omega (0) = 0$ *such that*

$$ \bigl\Vert kx + (1 - k)y \bigr\Vert ^{2} \le k\Vert x \Vert ^{2} + (1 - k)\Vert y \Vert ^{2} - k(1 - k)\omega \bigl( \Vert x - y \Vert \bigr) $$

*for* $k \in [0,1],x,y \in E$ *with* $\Vert x \Vert \le r$ *and* $\Vert y \Vert \le r$.

## Strong convergence theorems and experiments

### Strong convergence for infinite maximal monotone mappings and infinite weakly relatively non-expansive mappings

In this section, we suppose that the following conditions are satisfied:

1. *E* is a real uniformly convex and uniformly smooth Banach space and $J:E \to E^{*}$ is the normalized duality mapping;
2. $T_{i} \subset E \times E^{*}$ is maximal monotone and $S_{i}:E \to E$ is weakly relatively non-expansive for each $i \in N$;
3. $\{ s_{n,i}\}$ and $\{ \tau_{n}\}$ are two real number sequences in ($0, + \infty $) for $i,n \in N$. $\{ \alpha_{n}\}$ is a real number sequence in ($0,1$) for $n \in N$;
4. $\{ \varepsilon_{n}\}$ is the error sequence in *E*.

#### Algorithm 2.1

*Step* 1. Choose $u_{1},\varepsilon_{1} \in E$. Let $s_{1,i} \in (0, + \infty )$ for $i \in N$. $\alpha_{1} \in (0,1)$ and $\tau_{1} \in (0, + \infty )$. Set $n = 1$, and go to Step 2.

*Step* 2. Compute $v_{n,i} = (J + s_{n,i}T_{i})^{ - 1}J(u_{n} + \varepsilon_{n})$ and $w_{n,i} = J^{ - 1}[\alpha_{n}Ju_{n} + (1 - \alpha_{n})JS_{i}v_{n,i}]$ for $i \in N$. If $v_{n,i} = u_{n} + \varepsilon_{n}$ and $w_{n,i} = J^{ - 1}[\alpha_{n}Ju_{n} + (1 - \alpha_{n})J(u_{n} + \varepsilon_{n})]$ for all $i \in N$, then stop; otherwise, go to Step 3.

*Step* 3. Construct the sets $V_{n}$, $W_{n}$, and $U_{n}$ as follows:

$$\begin{aligned}& \textstyle\begin{cases} V_{1} = E, \\ V_{n + 1,i} = \{ z \in E: \langle v_{n,i} - z,J(u_{n} + \varepsilon_{n}) - Jv_{n,i} \rangle \ge 0\}, \\ V_{n + 1} = (\bigcap_{i = 1}^{\infty } V_{n + 1,i}) \cap V_{n}, \end{cases}\displaystyle \\& \textstyle\begin{cases} W_{1} = E, \\ W_{n + 1,i} = \{ z \in V_{n + 1,i}:\varphi (z,w_{n,i}) \le \alpha_{n} \varphi (z,u_{n}) + (1 - \alpha_{n})\varphi (z,v_{n,i})\}, \\ W_{n + 1} = (\bigcap_{i = 1}^{\infty } W_{n + 1,i}) \cap W_{n}, \end{cases}\displaystyle \end{aligned}$$

and

$$ U_{n + 1} = \bigl\{ z \in W_{n + 1}:\Vert u_{1} - z \Vert ^{2} \le \bigl\Vert P_{{W}_{n + 1}}(u_{1}) - u_{1} \bigr\Vert ^{2} + \tau_{n + 1} \bigr\} , $$

go to Step 4.

*Step* 4. Choose any element $u_{n + 1} \in U_{n + 1}$ for $n \in N$.

*Step* 5. Set $n = n + 1$, and return to Step 2.

#### Theorem 2.1

*If*, *in Algorithm *2.1, $v_{n,i} = u_{n} + \varepsilon_{n}$ *and* $w_{n,i} = J^{ - 1}[\alpha_{n}Ju_{n} + (1 - \alpha _{n})J(u_{n} + \varepsilon_{n})]$ *for all* $i \in N$, *then* $u_{n} + \varepsilon_{n} \in (\bigcap_{i = 1}^{\infty } N(T_{i})) \cap ( \bigcap_{i = 1}^{\infty } F(S_{i}))$.

#### Proof

Since $v_{n,i} = u_{n} + \varepsilon_{n}$, then from Step 2 in Algorithm 2.1, we know that $Jv_{n,i} + s_{n,i}T_{i}v_{n,i} = Jv _{n,i}$ for all $i \in N$, which implies that $s_{n,i}T_{i}v_{n,i} = 0$ for $i \in N$. Therefore, $u_{n} + \varepsilon_{n} \in \bigcap_{i = 1} ^{\infty } N(T_{i})$.

Since $w_{n,i} = J^{ - 1}[\alpha_{n}Ju_{n} + (1 - \alpha_{n})J(u_{n} + \varepsilon_{n})] = J^{ - 1}[\alpha_{n}Ju_{n} + (1 - \alpha_{n})JS _{i}v_{n,i}]$, then in view of Lemma 1.1 $v_{n,i} = S_{i}v_{n,i}$ for $i,n \in N$. Thus $v_{n,i} = u_{n} + \varepsilon_{n} \in \bigcap_{i = 1}^{\infty } F(S_{i})$, $n \in N$.

This completes the proof. □

#### Theorem 2.2

*Suppose* $(\bigcap_{i = 1}^{\infty } N(T_{i})) \cap (\bigcap_{i = 1}^{\infty } F(S_{i})) \ne \emptyset, \inf_{n}s _{n,i} > 0$ *for* $i \in N$, $0 < \sup_{n}\alpha_{n} < 1$, $\tau_{n} \to 0$, *and* $\varepsilon_{n} \to 0$, *as* $n \to \infty $. *Then the iterative sequence* $u_{n} \to y_{0} = P_{\bigcap_{n = 1}^{\infty } W _{n}} (u_{1})\in (\bigcap_{i = 1}^{\infty } N(T_{i})) \cap (\bigcap_{i = 1}^{\infty } F(S_{i}))$, *as* $n \to \infty $.

#### Proof

We split the proof into eight steps.

*Step* 1. $V_{n}$ is a nonempty subset of *E*.

In fact, we shall prove that $(\bigcap_{i = 1}^{\infty } N(T_{i})) \cap (\bigcap_{i = 1}^{\infty } F(S_{i})) \subset V_{n}$, which ensures that $V_{n} \ne \emptyset $.

For this, we shall use inductive method. Now, $\forall p \in ( \bigcap_{i = 1}^{\infty } N(T_{i})) \cap (\bigcap_{i = 1}^{\infty } F(S _{i}))$.

If $n = 1$, it is obvious that $p \in V_{1} = E$. Since $T_{i}$ is monotone, then

$$ \bigl\langle v_{1,i} - p, J(u_{1} + \varepsilon_{1})-Jv_{1,i} \bigr\rangle = \langle v_{1,i} - p,s_{1,i}T_{i}v_{1,i} - s_{1,i}T_{i}p \rangle \ge 0. $$

Thus $p \in V_{2,i}$, which ensures that $p \in V_{2}$.

Suppose the result is true for $n = k + 1$. Then, if $n = k + 2$, we have

$$ \bigl\langle v_{k + 1,i} - p, J(u_{k + 1} + \varepsilon_{k + 1})-Jv_{k+1,i}\bigr\rangle = \langle v_{k + 1,i} - p,s_{k + 1,i}T_{i}v_{k + 1,i} - s_{k + 1,i}T_{i}p \rangle \ge 0. $$

Then $p \in V_{k + 2,i}$, which ensures that $p \in V_{k + 2}$.

Therefore, by induction, $(\bigcap_{i = 1}^{\infty } N(T_{i})) \cap ( \bigcap_{i = 1}^{\infty } F(S_{i})) \subset V_{n}$ for $n \in N$.

*Step* 2. $W_{n}$ is a nonempty closed and convex subset of *E* for $n \in N$.

Since $\varphi (z,w_{n,i}) \le \alpha_{n} \varphi (z,u_{n}) + (1 - \alpha_{n})\varphi (z,v_{n,i})$ is equivalent to $\langle z,2\alpha_{n}Ju_{n} + 2(1 - \alpha_{n})Jv_{n,i} - 2Jw_{n,i} \rangle \leq \alpha_{n}\Vert u _{n} \Vert ^{2} + (1 - \alpha_{n})\Vert v_{n,i} \Vert ^{2} - \Vert w_{n,i} \Vert ^{2}$, then it is easy to see that $W_{n,i}$ is closed and convex for $i,n \in N$. Thus $W_{n}$ is closed and convex for $n \in N$.

Next, we shall use inductive method to show that $(\bigcap_{i = 1} ^{\infty } N(T_{i})) \cap (\bigcap_{i = 1}^{\infty } F(S_{i})) \subset W_{n}$ for $n \in N$, which ensures that $W_{n} \ne \emptyset $ for $n \in N$.

In fact, $\forall p \in (\bigcap_{i = 1}^{\infty } N(T_{i})) \cap ( \bigcap_{i = 1}^{\infty } F(S_{i}))$.

If $n = 1$, it is obvious that $p \in W_{1} = E$. Then, from the definition of weakly relatively non-expansive mappings, we have

$$\begin{aligned} \varphi (p,w_{n,i}) &\le \alpha_{1}\varphi (p,u_{1}) + (1 - \alpha_{1}) \varphi (p,S_{i}v_{1,i}) \\ &\le \alpha_{1}\varphi (p,u_{1}) + (1 - \alpha_{1}) \varphi (p,v_{1,i}). \end{aligned}$$

Combining this with Step 1, we know that $p \in W_{2,i}$ for $i \in N$. Therefore, $p \in W_{2}$.

Suppose the result is true for $n = k + 1$. Then, if $n = k + 2$, we know from Step 1 that $p \in V_{k + 2,i}$ for $i,k \in N$. Moreover,

$$\begin{aligned} \varphi (p,w_{k + 1,i}) &\le \alpha_{k + 1}\varphi (p,u_{k + 1}) + (1 -\alpha_{k+ 1})\varphi (p,S_{i}v_{k + 1,i}) \\ & \le \alpha_{k + 1}\varphi (p,u_{k + 1}) + (1 -\alpha_{k + 1}) \varphi (p,v_{k + 1,i}), \end{aligned}$$

which implies that $p \in W_{k + 2,i}$, and then $p \in (\bigcap_{i = 1}^{\infty } W_{k + 2,i}) \cap W_{k +1} = W_{k + 2}$. Therefore, by induction,

$$\begin{aligned} \emptyset \ne \Biggl(\bigcap_{i = 1}^{\infty } N(T_{i}) \Biggr) \cap \Biggl(\bigcap_{i = 1}^{\infty } F(S_{i}) \Biggr) \subset W_{n} \quad\mbox{for } n \in N. \end{aligned}$$

*Step* 3. Set $y_{n} = P_{W_{n + 1}}(u_{1})$. Then $y_{n} \to y_{0} = P_{\bigcap_{n = 1}^{\infty } W_{n}} (u_{1})$, as $n \to \infty $.

From the construction of $W_{n}$ in Step 3 of Algorithm 2.1, $W_{n + 1} \subset W_{n}$ for $n \in N$. Lemma 1.10 implies that $\lim W_{n}$ exists and $\lim W_{n} = \bigcap_{n = 1}^{\infty } W_{n} \ne \emptyset $. Since *E* has Property (H), then Lemma 1.11 implies that $y_{n} \to y_{0} = P_{\bigcap_{n = 1}^{\infty } W_{n}} (u_{1})$, as $n \to \infty $.

*Step* 4. $\{ u_{n}\}$ is well defined.

It suffices to show that $U_{n} \ne \emptyset $. From the definitions of $P_{W_{n + 1}}(u_{1})$ and infimum, we know that for $\tau_{n + 1}$ there exists $b_{n} \in W_{n + 1}$ such that

$$ \Vert u_{1} - b_{n} \Vert ^{2} \le \Bigl(\inf_{z \in W_{n + 1}}\Vert u_{1} - z \Vert \Bigr)^{2} + \tau_{n + 1} = \bigl\Vert P_{W_{n + 1}}(u_{1}) - u _{1} \bigr\Vert ^{2} + \tau_{n + 1}. $$

This ensures that $U_{n + 1} \ne \emptyset $ for $n \to \infty $.

*Step* 5. $u_{n + 1} - y_{n} \to 0$ as $n \to \infty $.

Since $u_{n + 1} \in U_{n + 1} \subset W_{n + 1}$, then in view of Lemma 1.13 and the fact that $W_{n}$ is convex, we have, for $\forall k \in (0,1)$,

$$\begin{aligned} \Vert y_{n} - u_{1} \Vert ^{2} &\le \bigl\Vert ky_{n} + (1 - k)u_{n + 1} - u_{1} \bigr\Vert ^{2} \\ &\le k\Vert y_{n} - u_{1} \Vert ^{2} + (1 - k)\Vert u_{n + 1} - u_{1} \Vert ^{2} - k(1 - k) \omega \bigl(\Vert y _{n} - u_{n + 1} \Vert \bigr). \end{aligned}$$

Therefore,

$$\begin{aligned} k\omega \bigl(\Vert y_{n} - u_{n + 1} \Vert \bigr) \le \Vert u_{n + 1} -u_{1} \Vert ^{2} - \Vert y_{n} -u_{1} \Vert ^{2} \le \tau_{n + 1}. \end{aligned}$$

Letting $k \to 1$, then $y_{n} - u_{n + 1} \to 0$ as $n \to \infty $. Since $y_{n} \to y_{0}$, then $u_{n} \to y_{0}$, as $n \to \infty $.

*Step* 6. $u_{n} - v_{n,i} \to 0$ for $i \in N$, as $n \to \infty $.

Since $y_{n + 1} \in W_{n + 2} \subset W_{n + 1} \subset V_{n + 1}$, then

$$\begin{aligned} 0 &\le 2 \bigl\langle v_{n,i} - y_{n + 1},J(u_{n} + \varepsilon_{n}) - Jv _{n,i} \bigr\rangle \\ &= 2 \bigl\langle y_{n + 1} - v_{n,i},Jv_{n,i} - J(u _{n} + \varepsilon_{n}) \bigr\rangle \\ &= \varphi (y_{n + 1},u_{n} + \varepsilon_{n}) - \varphi (y_{n + 1},v_{n,i}) - \varphi (v_{n,i},u_{n} + \varepsilon_{n}) \\ &\le \varphi (y_{n + 1},u_{n} + \varepsilon_{n}) - \varphi (v_{n,i},u_{n} + \varepsilon_{n}). \end{aligned}$$

Thus, by using Step 5 and by letting $\varepsilon_{n} \to 0$, we have

$$\begin{aligned} \varphi (v_{n, i},u_{n} + \varepsilon_{n}) &\le \varphi (y_{n + 1},u_{n} + \varepsilon_{n}) \\ &= \varphi (y_{n + 1},y_{n}) + \varphi (y_{n},u_{n} + \varepsilon_{n}) + 2 \bigl\langle y_{n + 1} - y_{n},Jy_{n} - J(u_{n} + \varepsilon_{n}) \bigr\rangle \\ &\le \bigl(\Vert y_{n + 1} \Vert \Vert Jy_{n + 1} - Jy_{n} \Vert + \Vert y_{n + 1} - y_{n} \Vert \Vert y_{n} \Vert \bigr) \\ &\quad {}+ \bigl(\Vert y_{n} \Vert \bigl\Vert Jy_{n} - J(u_{n} + \varepsilon_{n}) \bigr\Vert + \Vert y_{n + 1} - u_{n} - \varepsilon_{n} \Vert \Vert u_{n} + \varepsilon_{n} \Vert \bigr) \\ &\quad {}+ 2\Vert y_{n + 1} - y_{n} \Vert \bigl\Vert Jy_{n} - J(u_{n} + \varepsilon_{n}) \bigr\Vert \to 0, \end{aligned}$$

as $n \to \infty $. Using Lemma 1.12, $v_{n,i} - u_{n} - \varepsilon _{n} \to 0$ for $i \in N$, as $n \to \infty $. Since $\varepsilon_{n} \to 0$, then $v_{n,i} - u_{n} \to 0$ for $i \in N$, as $n \to \infty $. Since $u_{n} \to y_{0}$, then $v_{n,i} \to y_{0}$ for $i \in N$, as $n \to \infty $.

*Step* 7. $w_{n,i} - u_{n} \to 0$ for $i \in N$, as $n \to \infty $.

Since $u_{n + 1} \in U_{n + 1} \subset W_{n + 1}$, then noticing Steps 5 and 6,

$$\begin{aligned} \varphi (u_{n + 1},w_{n,i}) \le \alpha_{n}\varphi (u_{n + 1},u_{n}) + (1 - \alpha_{n})\varphi (u_{n + 1},v_{n,i}) \to 0, \end{aligned}$$

as $n \to \infty $. Lemma 1.12 implies that $u_{n + 1} - w_{n,i} \to 0$, as $n \to \infty $. Since $u_{n} \to y_{0}$, then $w_{n,i} \to y_{0}$ for $i \in N$, as $n \to \infty $.

*Step* 8. $y_{0} = P_{\bigcap_{n = 1}^{\infty } W_{n}} (u_{1}) \in (\bigcap_{i = 1}^{\infty } N(T_{i})) \cap (\bigcap_{i = 1}^{\infty } F(S _{i}))$.

Since $v_{n,i} = (J + s_{n,i}T_{i})^{ - 1}J(u_{n} + \varepsilon_{n})$, then $Jv_{n,i} + s_{n,i}T_{i}v_{n,i} = J(u_{n} + \varepsilon_{n})$. Since $v_{n,i} \to y_{0}$, $u_{n} \to y_{0}$, $\varepsilon_{n} \to 0$ and $\inf_{n}s_{n,i} > 0$, then $T_{i}v_{n,i} \to 0$ for $i \in N$, as $n \to \infty $. Using Lemma 1.7, $y_{0} \in \bigcap_{i = 1}^{\infty } N(T_{i})$.

Since $w_{n,i} = J^{ - 1}[\alpha_{n}Ju_{n} + (1 - \alpha_{n})JS_{i}v_{n,i}]$, then in view of Lemma 1.1, $S_{i}v_{n,i} \to y_{0}$, as $n \to \infty $. Lemma 1.6 implies that $y_{0} \in \bigcap_{i = 1}^{\infty } F(S_{i})$.

This completes the proof. □

#### Corollary 2.3

*If* $i \equiv 1$, *denote by* *T* *the maximal monotone mapping and by* *S* *the weakly relatively non*-*expansive mapping*, *then Algorithm *2.1 *reduces to the following*:

$$\begin{aligned} \textstyle\begin{cases} u_{1} \in E,\quad \varepsilon_{1} \in E, \\ v_{n} = (J + s_{n}T)^{ - 1}J(u_{n} + \varepsilon_{n}), \\ w_{n} = J^{ - 1}[\alpha_{n}Ju_{n} + (1 - \alpha_{n})JSv_{n}], \\ V_{1} = W_{1} = E, \\ V_{n + 1} = \{ z \in E: \langle v_{n} - z,J(u_{n} + \varepsilon _{n}) - Jv_{n} \rangle \ge 0\} \cap V_{n}, \\ W_{n + 1} = \{ z \in V_{n + 1}:\varphi (z,w_{n}) \le \alpha_{n}\varphi (z,u _{n}) + (1 - \alpha_{n})\varphi (z,v_{n})\} \cap W_{n}, \\ U_{n + 1} = \{ z \in W_{n + 1}:\Vert u_{1} - z \Vert ^{2} \le \Vert P_{W_{n + 1}}(u_{1}) - u_{1} \Vert ^{2} + \tau_{n + 1}\}, \\ u_{n + 1} \in U_{n + 1},\quad n \in N, \end{cases}\displaystyle \end{aligned}$$

*where* $\{ \varepsilon_{n}\} \subset E$, $\{ s_{n}\} \subset (0,\infty )$, $\{ \tau_{n}\} \subset (0,\infty )$, *and* $\{ \alpha_{n}\} \subset (0,1)$. *Then*

1. *Similar to Theorem *2.1, *if* $v_{n} = u_{n} + \varepsilon_{n}$ *and* $w_{n} = J^{ - 1}[\alpha_{n}Ju_{n} + (1 - \alpha_{n})J(u_{n} + \varepsilon_{n})]$ *for all* $n \in N$, *then* $u_{n} + \varepsilon_{n} \in N(T) \cap F(S)$.
2. *Suppose that* *E*, $\{ \varepsilon_{n}\}$, $\{ \tau_{n}\}$, *and* $\{ \alpha_{n}\}$ *satisfy the same conditions as those in Theorem *2.2. *If* $N(T) \cap F(S) = \emptyset $ *and* $\inf_{n}s_{n} > 0$, *then the iterative sequence* $u_{n} \to y_{0} = P_{\bigcap_{n= 1}^{\infty } W_{n}} (u_{1}) \in N(T) \cap F(S)$, *as* $n \to \infty $.

#### Algorithm 2.2

Only doing the following changes in Algorithm 2.1, we get Algorithm 2.2:

$$ w_{n,i} = J^{ - 1} \bigl[\alpha_{n}Ju_{1} + (1 - \alpha_{n})JS_{i}v_{n,i} \bigr] \quad \mbox{for all } i \in N, $$

and

$$ \textstyle\begin{cases} W_{1} = E, \\ W_{n + 1, i} = \{ z \in V_{n + 1, i}:\varphi (z,w_{n,i}) \le \alpha_{n}\varphi (z,u _{1}) + (1 - \alpha_{n})\varphi (z,v_{n,i})\}, \\ W_{n + 1} = (\bigcap_{i = 1}^{\infty } W_{n + 1,i} )\cap W_{n}. \end{cases} $$

#### Theorem 2.4

*If*, *in Algorithm *2.2, $v_{n,i} = u_{n} + \varepsilon_{n}$ *and* $w_{n,i} = J^{ - 1}[\alpha_{n}Ju_{1} + (1 - \alpha_{n})J(u_{n} + \varepsilon_{n})]$ *for all* $i \in N$, *then* $u_{n} + \varepsilon_{n} \in (\bigcap_{i = 1}^{\infty } N(T_{i} )) \cap (\bigcap_{i = 1}^{\infty } F(S_{i} ))$.

#### Proof

Similar to Theorem 2.1, the result follows. □

#### Theorem 2.5

*We only change the condition that* $0 < \sup_{n} \alpha_{n} < 1$ *in Theorem *2.2 *by* $\alpha_{n} \to 0$, *as* $n \to \infty $. *Then the iterative sequence* $u_{n} \to y_{0} = P_{\bigcap _{n = 1}^{\infty } W_{n}} (u_{1}) \in (\bigcap_{i = 1}^{\infty } N(T_{i} )) \cap (\bigcap_{i = 1}^{\infty } F(S_{i} ))$, *as* $n \to \infty $.

#### Proof

Copy Steps 1, 3, 4, 5, and 6 in Theorem 2.2 and make slight changes in the following steps.

*Step* 2. $W_{n}$ is a nonempty closed and convex subset of *E* for $n \in N$.

Since $\varphi (z,w_{n,i}) \le \alpha_{n}\varphi (z,u_{1}) + (1 - \alpha _{n})\varphi (z,v_{n,i})$ is equivalent to $\langle z,2\alpha_{n}Ju _{1} + 2(1 - \alpha_{n})Jv_{n,i} - 2Jw_{n,i} \rangle \le \alpha _{n}\Vert u_{1} \Vert ^{2} + (1 - \alpha_{n})\Vert v_{n,i} \Vert ^{2} - \Vert w_{n,i} \Vert ^{2}$, then it is easy to see that $W_{n,i}$ is closed and convex for $i,n \in N$. Thus $W_{n}$ is closed and convex for $n \in N$.

Next, we shall use inductive method to show that $(\bigcap_{i = 1} ^{\infty } N(T_{i} )) \cap (\bigcap_{i = 1}^{\infty } F(S_{i} )) \subset W_{n}$ for $n \in N$, which ensures that $W_{n} \ne \emptyset $ for $n \in N$.

In fact, $\forall p \in (\bigcap_{i = 1}^{\infty } N(T_{i} )) \cap ( \bigcap_{i = 1}^{\infty } F(S_{i} ))$.

If $n = 1$, it is obvious that $p \in W_{1} = E$. Then, from the definition of weakly relatively non-expansive mappings, we have

$$\begin{aligned} \varphi (p,w_{1,i}) &\le \alpha_{1}\varphi (p,u_{1}) + (1 - \alpha_{1}) \varphi (p,S_{i}v_{1,i}) \\ &\le \alpha_{1}\varphi (p,u_{1}) + (1 - \alpha_{1}) \varphi (p,v_{1,i}). \end{aligned}$$

Combining this with Step 1, we know that $p \in W_{2,i}$ for $i \in N$. Therefore, $p \in W_{2}$.

Suppose the result is true for $n = k + 1$. Then, if $n = k + 2$, we know from Step 1 that $p \in V_{k + 2,i}$ for $i,k \in N$. Moreover,

$$\begin{aligned} \varphi (p,w_{k + 1,i}) &\le \alpha_{k + 1}\varphi (p,u_{1}) + (1 - \alpha_{k + 1})\varphi (p,S_{i}v_{k + 1,i}) \\ &\le \alpha_{k + 1}\varphi (p,u _{1}) + (1 - \alpha_{k + 1})\varphi (p,v_{k + 1,i}), \end{aligned}$$

which implies that $p \in W_{k + 2,i}$ and then $p \in (\bigcap_{i = 1}^{\infty } W_{k + 2,i} ) \cap W_{k + 1} = W_{k + 2}$. Therefore, by induction, $\emptyset \ne (\bigcap_{i = 1}^{\infty } N(T_{i} )) \cap (\bigcap_{i = 1}^{\infty } F(S_{i} )) \subset W_{n}$ for $n \in N$.

*Step* 7. $w_{n,i} - u_{n} \to 0$ for $i \in N$, as $n \to \infty $.

Since $u_{n + 1} \in U_{n + 1} \subset W_{n + 1}$, then in view of the facts that $\alpha_{n} \to 0$ and Step 6,

$$ \varphi (u_{n + 1},w_{n,i}) \le \alpha_{n}\varphi (u_{n + 1},u_{1}) + (1 - \alpha_{n}) \varphi (u_{n + 1},v_{n,i}) \to 0, $$

as $n \to \infty $, for $i \in N$. Lemma 1.12 implies that $w_{n,i} - u_{n} \to 0$ for $i \in N$, as $n \to \infty $.

*Step* 8. $y_{0} = P_{\bigcap_{n = 1}^{\infty } W_{n}} (u_{1}) \in ( \bigcap_{i = 1}^{\infty } N(T_{i} )) \cap (\bigcap_{i = 1}^{\infty } F(S _{i} ))$.

In the same way as Step 8 in Theorem 2.2, we have $y_{0} \in \bigcap_{i = 1}^{\infty } N(T_{i})$. Since $w_{n,i} = J^{ - 1}[\alpha _{n}Ju_{1} + (1 - \alpha_{n})JS_{i}v_{n,i}]$, then $S_{i}v_{n,i} \to y _{0}$, as $n \to \infty $. Thus in view of Lemma 1.6, $y_{0} \in \bigcap_{i = 1}^{\infty } F(S_{i})$.

This completes the proof. □

#### Corollary 2.6

*If* $i \equiv 1$, *denote by* *T* *the maximal monotone mapping and by* *S* *the weakly relatively non*-*expansive mapping*, *then Algorithm *2.2 *reduces to the following*:

$$ \textstyle\begin{cases} u_{1} \in E,\quad \varepsilon_{1} \in E, \\ v_{n} = (J + s_{n}T)^{ - 1}J(u_{n} + \varepsilon_{n}), \\ w_{n} = J^{ - 1}[\alpha_{n}Ju_{1} + (1 - \alpha_{n})JSv_{n}], \\ V_{1} = W_{1} = E, \\ V_{n + 1} = \{ z \in E: \langle v_{n} - z,J(u_{n} + \varepsilon _{n}) - Jv_{n} \rangle \ge 0\} \cap V_{n}, \\ W_{n + 1} = \{ z \in V_{n + 1}:\varphi (z,w_{n}) \le \alpha_{n}\varphi (z,u_{1})+ (1 - \alpha_{n})\varphi (z,v_{n})\} \cap W_{n}, \\ U_{n + 1} = \{ z \in W_{n + 1}:\Vert u_{1} - z \Vert ^{2} \le \Vert P_{W_{n + 1}}(u_{1}) - u_{1} \Vert ^{2} + \tau_{n + 1}\}, \\ u_{n + 1} \in U_{n + 1},\quad n \in N, \end{cases} $$

*where* $\{ \varepsilon_{n}\} \subset E$, $\{ s_{n}\} \subset (0,\infty )$, $\{ \tau_{n}\} \subset (0,\infty )$ *and* $\{ \alpha_{n}\} \subset (0,1)$. *Then*

1. *Similar to Theorem *2.4, *if* $v_{n} = u_{n} + \varepsilon_{n}$ *and* $w_{n} = J^{ - 1}[\alpha_{n}Ju_{1} + (1 - \alpha_{n})J(u_{n} + \varepsilon_{n})]$, *then* $u_{n} + \varepsilon_{n} \in N(T) \cap F(S)$ *for all* $n \in N$.
2. *Suppose that* *E*, $\{ \varepsilon_{n}\}$, $\{ \tau_{n}\}$, *and* $\{ \alpha_{n}\}$ *satisfy the same conditions as those in Theorem *2.5. *If* $N(T) \cap F(S) = \emptyset $ *and* $\inf_{n}s_{n} > 0$, *then the iterative sequence* $u_{n} \to y_{0} = P_{\bigcap_{n = 1}^{\infty } W_{n}} (u_{1})\in N(T) \cap F(S)$ *as* $n \to \infty $.

#### Remark 2.7

Compared to the existing related work, e.g., [12–14], strongly relatively non-expansive mappings are extended to weakly relatively non-expansive mappings. Moreover, in our paper, the discussion on this topic is extended to the case of infinite maximal monotone mappings and infinite weakly relatively non-expansive mappings.

#### Remark 2.8

Calculating the generalized projection $\Pi_{H_{n} \cap V_{n} \cap W_{n}}(x_{1})$ in [12] or $\Pi_{H_{n} \cap V_{n}}(x_{1})$ in [13] is replaced by calculating the projection $P_{W_{n + 1}}(u_{1})$ in Step 3 in our Algorithms 2.1 and 2.2, which makes the computation easier.

#### Remark 2.9

A new proof technique for finding the limit $y_{0} = P_{\bigcap_{n = 1}^{\infty } W_{n}} (u_{1})$ is employed in our paper by examining the properties of the projective sets $W_{n}$ sufficiently, which is quite different from that for finding the limit $\Pi_{N(T) \cap F(S)}(x_{1})$ in [12] or $\Pi_{N(A) \cap F(S) \cap F(T)}(x _{1})$ in [13].

#### Remark 2.10

Theoretically, the projection is easier for calculating than the generalized projection in a general Banach space since the generalized projection involves a Lyapunov functional. In this sense, iterative algorithms constructed in our paper are new and more efficient.

### Special cases in Hilbert spaces and computational experiments

#### Corollary 2.11

*If* *E* *reduces to a Hilbert space H*, *then iterative Algorithm *2.1 *becomes the following one*:

2.1 $$\begin{aligned} \textstyle\begin{cases} u_{1} \in H,\quad \varepsilon_{1} \in H, \\ v_{n,i} = (I + s_{n,i}T_{i})^{ - 1}(u_{n} + \varepsilon_{n}), \\ w_{n,i} = \alpha_{n}u_{n} + (1 - \alpha_{n})S_{i}v_{n,i}, \\ V_{1} = W_{1} = H, \\ V_{n + 1,i} = \{ z \in H: \langle v_{n,i} - z,u_{n} + \varepsilon _{n} - v_{n,i} \rangle \ge 0\}, \\ V_{n + 1} = (\bigcap_{i = 1}^{\infty } V_{n + 1,i} ) \cap V_{n}, \\ W_{n + 1,i} = \{ z \in V_{n + 1,i}:\Vert z - w_{n,i} \Vert ^{2}\le \alpha_{n}\Vert z - u_{n} \Vert ^{2} + (1 - \alpha_{n})\Vert z - v_{n,i} \Vert ^{2}\}, \\ W_{n + 1} = (\bigcap_{i = 1}^{\infty } W_{n + 1,i} ) \cap W_{n}, \\ U_{n + 1} = \{ z \in W_{n + 1}:\Vert u_{1} - z \Vert ^{2} \le \Vert P_{W_{n + 1}}(u_{1}) - u_{1} \Vert ^{2} + \tau_{n + 1}\}, \\ u_{n + 1} \in U_{n + 1},\quad n \in N. \end{cases}\displaystyle \end{aligned}$$

The results of Theorems 2.1 and 2.2 are true for this special case.

#### Corollary 2.12

*If* *E* *reduces to a Hilbert space H*, *then iterative Algorithm *2.2 *becomes the following one*:

2.2 $$ \textstyle\begin{cases} u_{1} \in H,\quad \varepsilon _{1} \in H, \\ v_{n,i} = (I + s_{n,i}T_{i})^{ - 1}(u_{n} + \varepsilon_{n}), \\ w_{n,i} = \alpha_{n}u_{1} + (1 - \alpha_{n})S_{i}v_{n,i}, \\ V_{1} = W_{1} = H, \\ V_{n + 1,i} = \{ z \in H: \langle v_{n,i} - z,u_{n} + \varepsilon_{n} - v_{n,i} \rangle \ge 0\}, \\ V_{n + 1} = (\bigcap_{i = 1}^{\infty } V_{n + 1,i} ) \cap V_{n},\\ W_{n + 1,i} = \{ z \in V_{n + 1,i}:\Vert z - w_{n,i} \Vert ^{2} \le \alpha_{n}\Vert z - u_{1} \Vert ^{2} + (1 - \alpha_{n})\Vert z - v_{n,i} \Vert ^{2}\}, \\ W_{n + 1} = (\bigcap_{i = 1}^{\infty } W_{n + 1,i} ) \cap W_{n}, \\ U_{n + 1} = \{ z \in W_{n + 1}:\Vert u_{1} - z \Vert ^{2} \le \Vert P_{W_{n + 1}}(u_{1}) - u_{1} \Vert ^{2} + \tau_{n + 1}\}, \\ u_{n + 1} \in U_{n + 1},\quad n \in N. \end{cases} $$

The results of Theorems 2.4 and 2.5 are true for this special case.

#### Corollary 2.13

*If*, *further* $i \equiv 1$, *then* (2.1) *and* (2.2) *reduce to the following two cases*:

2.3 $$ \textstyle\begin{cases} u_{1} \in H,\quad \varepsilon _{1} \in H, \\ v_{n} = (I + s_{n}T)^{ - 1}(u_{n} + \varepsilon_{n}), \\ w_{n} = \alpha_{n}u_{n} + (1 - \alpha_{n})Sv_{n}, \\ V_{1} = W_{1} = H, \\ V_{n + 1} = \{ z \in H: \langle v_{n} - z,u_{n} + \varepsilon_{n} - v_{n} \rangle \ge 0\} \cap V_{n}, \\ W_{n + 1} = \{ z \in V_{n + 1}:\Vert z - w_{n} \Vert ^{2} \le \alpha_{n}\Vert z - u_{n} \Vert ^{2} + (1 - \alpha_{n})\Vert z - u_{n} \Vert ^{2}\} \cap W_{n}, \\ U_{n + 1} = \{ z \in W_{n + 1}:\Vert u_{1} - z \Vert ^{2} \le \Vert P_{W_{n + 1}}(u_{1}) - u_{1} \Vert ^{2} + \tau_{n + 1}\}, \\ u_{n + 1} \in U_{n + 1},\quad n \in N, \end{cases} $$

*and*

2.4 $$ \textstyle\begin{cases} u_{1} \in H,\quad \varepsilon _{1} \in H, \\ v_{n} = (I + s_{n}T)^{ - 1}(u_{n} + \varepsilon_{n}), \\ w_{n} = \alpha_{n}u_{1} + (1 - \alpha_{n})Sv_{n}, \\ V_{1} = W_{1} = H, \\ V_{n + 1} = \{ z \in H: \langle v_{n} - z,u_{n} + \varepsilon_{n} - v_{n} \rangle \ge 0\} \cap V_{n}, \\ W_{n + 1} = \{ z \in V_{n + 1}:\Vert z - w_{n} \Vert ^{2} \le \alpha_{n}\Vert z - u_{1} \Vert ^{2} + (1 - \alpha_{n})\Vert z - u_{n} \Vert ^{2}\} \cap W_{n}, \\ U_{n + 1} = \{ z \in W_{n + 1}:\Vert u_{1} - z \Vert ^{2} \le \Vert P_{W_{n + 1}}(u_{1}) - u_{1} \Vert ^{2} + \tau_{n + 1}\}, \\ u_{n + 1} \in U_{n + 1},\quad n \in N. \end{cases} $$

The results of Corollaries 2.3 and 2.6 are true for the special cases, respectively.

#### Remark 2.14

Take $H = ( - \infty, + \infty )$, $Tx = 2x$, and $Sx = x$ for $x \in ( - \infty, + \infty )$. Let $\varepsilon_{n} = \alpha_{n} = \tau_{n} = \frac{1}{n}$ and $s_{n} = 2^{n - 1}$ for $n \in N$. Then *T* is maximal monotone and *S* is weakly relatively non-expansive. Moreover, $N(T) \cap F(S) = \{ 0\}$.

#### Remark 2.15

Taking the example in Remark 2.14 and choosing the initial value $u_{1} = 1 \in ( - \infty, + \infty )$, we can get an iterative sequence $\{ u_{n}\}$ by algorithm (2.3) in the following way:

2.5 $$\begin{aligned} \textstyle\begin{cases} u_{1} = 1 \in ( - \infty, + \infty ), \\ u_{n + 1} = \frac{u_{1} + v_{n} - \sqrt{(u_{1} - v_{n})^{2} + \tau_{n + 1}}}{2},\quad n \in N, \end{cases}\displaystyle \end{aligned}$$

where $v_{n} = \frac{u_{n} + \varepsilon_{n}}{1 + 2s_{n}}$, $n \in N$. Moreover, $u_{n} \to 0 \in N(T) \cap F(S)$, as $n \to \infty $.

#### Proof

We can easily see from iterative algorithm (2.3) that

2.6 $$ v_{n} = \frac{u_{n} + \varepsilon_{n}}{1 + 2s_{n}}\quad \mbox{for } n \in N $$

and

2.7 $$ w_{n} = \alpha_{n}u_{n} + (1 - \alpha_{n})v_{n}\quad \mbox{for } n \in N. $$

To analyze the construction of set $W_{n}$, we notice that $\vert z - w_{n} \vert ^{2} \le \alpha_{n}\vert z - u_{n} \vert ^{2} + (1 - \alpha_{n})\vert z - v_{n} \vert ^{2}$ is equivalent to

2.8 $$ \bigl[2\alpha_{n}u_{n} + 2(1 - \alpha_{n})v_{n} - 2w_{n} \bigr]z \le \alpha_{n}u _{{n}}^{2} + (1 - \alpha_{n})v_{n}^{2} - w_{n}^{2}. $$

In view of (2.7), compute the left-hand side of (2.8):

2.9 $$\begin{aligned} &\bigl[2\alpha_{n}u_{n} + 2(1 - \alpha_{n})v_{n}- 2w_{n} \bigr]z \\ &\quad = \bigl[2\alpha_{n}u _{n} + 2(1 - \alpha_{n})v_{n} - 2\alpha_{n}u_{n} - 2(1 - \alpha_{n})v _{n} \bigr]z \\ &\quad \equiv 0\quad \mbox{for }n \in N. \end{aligned}$$

Meanwhile, compute the right-hand side of (2.8):

2.10 $$\begin{aligned} &\alpha_{n}u_{{n}}^{2} + (1 - \alpha_{n})v_{n}^{2} - w_{n}^{2} \\ &\quad = \alpha_{n}u_{{n}}^{2} + (1 - \alpha_{n})v_{n}^{2} - \alpha_{n}^{2}u _{n}^{2} - 2\alpha_{n}(1 - \alpha_{n})u_{n}v_{n} - (1 - \alpha_{n})^{2}v _{n}^{2} \\ &\quad = \alpha_{n}(1 - \alpha_{n})u_{n}^{2} + \alpha_{n}(1 - \alpha_{n})v _{n}^{2} - 2\alpha_{n}(1 - \alpha_{n})u_{n}v_{n} \\ &\quad = \alpha_{n}(1 - \alpha_{n}) (u_{n} - v_{n})^{2}\quad \mbox{for } n \in N. \end{aligned}$$

Using (2.8)–(2.10), we get

2.11 $$ W_{n + 1} = V_{n + 1} \cap W_{n}\quad \mbox{for } n \in N. $$

Next, we shall use inductive method to show that

2.12 $$\begin{aligned} \textstyle\begin{cases} 0 < v_{n + 1} < v_{n} < 1, \\ v_{n} > \frac{1}{2^{n + 1}(n + 1)}, \\ V_{n + 1} = ( - \infty,v_{n}], \\ W_{n + 1} = V_{n + 1}, \\ U_{n + 1} = [u_{1} - \sqrt{(u_{1} - v_{n})^{2} + \tau_{n + 1}},v _{n}], \\ \textrm{we may choose } u_{n + 1} = \frac{u_{1} + v _{n} - \sqrt{(u_{1} - v_{n})^{2} + \tau_{n + 1}}}{2}\quad \mbox{for } n\in N. \end{cases}\displaystyle \end{aligned}$$

In fact, if $n = 1$, then $v_{1} = \frac{u_{1} + \varepsilon_{1}}{1 + 2s_{1}} = \frac{2}{3}$, thus $V_{2} = ( - \infty,v_{1}] \cap V_{1} = ( - \infty,v_{1}]$. From (2.11), $W_{2} = V_{2} \cap W_{1} = V_{2}$. And then $P_{W_{2}}(u_{1}) = v_{1} = \frac{2}{3}$. So we have

$$\begin{aligned} U_{2} &= \bigl\{ z \in W_{2}:\vert u_{1} - z\vert \le \sqrt{ \bigl\vert P_{W _{2}}(u_{1}) - u_{1} \bigr\vert ^{2} + \tau_{2}} \bigr\} \\ &= \biggl[1 - \sqrt{\frac{1}{9} + \frac{1}{2}}, 1 + \sqrt{ \frac{1}{9} + \frac{1}{2}} \biggr] \cap \biggl( - \infty,\frac{2}{3} \biggr] \\ &= \biggl[1 - \sqrt{\frac{1}{9} + \frac{1}{2}}, \frac{2}{3} \biggr] \\ &= \bigl[u_{1} - \sqrt{(u_{1} - v_{1})^{2} + \tau_{2}},v_{1} \bigr]. \end{aligned}$$

Therefore, we may choose $u_{2} \in U_{2}$ as follows:

$$ u_{2} = \frac{u_{1} + v_{1} - \sqrt{(u_{1} - v_{1})^{2} + \tau_{2}}}{2}. $$

From (2.6), $v_{2} = \frac{u_{2} + \varepsilon_{2}}{1 + 2s_{2}} = \frac{4}{15} - \frac{\sqrt{22}}{60}$. Then $0 < v_{2} < v_{1} < 1$. And it is easy to see $v_{1} > \frac{1}{2^{1 + 1}(1 + 1)}$. Thus (2.12) is true for $n + 1$.

Suppose (2.12) is true for $n = k$, that is,

$$\begin{aligned} \textstyle\begin{cases} 0 < v_{k + 1} < v_{k} < 1, \\ v_{k} > \frac{1}{2^{k + 1}(k + 1)}, \\ V_{k + 1} = ( - \infty,v_{k}], \\ W_{k + 1} = V_{k + 1}, \\ U_{k + 1} = [u_{1} - \sqrt{(u_{1} - v_{k})^{2} + \tau_{k + 1}},v _{k}], \\ \mbox{we may choose } u_{k + 1} = \frac{u_{1} + v_{k} - \sqrt{(u_{1} - v_{k})^{2} + \tau_{k + 1}}}{2}. \end{cases}\displaystyle \end{aligned}$$

Then, for $n = k + 1$, we first analyze the set $V_{k + 2}$.

Note that $u_{k + 1} + \varepsilon_{k + 1} - v_{k + 1} = (1 + 2s_{k + 1})v_{k + 1} - v_{k + 1} = 2s_{k + 1}v_{k + 1} > 0$, then $\langle v_{k + 1} - z,u_{k + 1} + \varepsilon_{k + 1} - v_{k + 1} \rangle \ge 0$ is equivalent to $z \le v_{k + 1}$. Then

$$ V_{k + 2} = ( - \infty,v_{k + 1}] \cap V_{k + 1}= ( -\infty,v_{k +1}] \cap ( - \infty,v_{k}] = ( - \infty ,v_{k + 1}]. $$

From (2.11),

$$ W_{k+ 2} =V_{k + 2} \cap W_{k + 1} = ( -\infty,v_{k + 1}] \cap V_{k + 1} = V_{k + 2}. $$

Now, we analyze set $U_{k + 2}$.

Since $0 < v_{k + 1} < 1 = u_{1}$, then $P_{W_{k + 2}}(u_{1}) = v_{k + 1}$. Thus $\vert u_{1} - z \vert \le \sqrt{ \vert P_{W_{k + 2}}(u _{1}) - u_{1} \vert ^{2} + \tau_{k + 2}}$ is equivalent to $u_{1} - \sqrt{(u_{1} - v_{k + 1})^{2} + \tau_{k + 2}} \le z \le u _{1} + \sqrt{(u_{1} - v_{k + 1})^{2} + \tau_{k + 2}}$.

It is easy to check that $u_{1} + \sqrt{(u_{1} - v_{k + 1})^{2} + \tau_{k + 2}} > 1 > v_{k + 1}$, and $u_{1} - \sqrt{(u_{1} - v_{k + 1})^{2} + \tau_{k + 2}} < u_{1} - (u_{1} - v_{k + 1}) = v_{k + 1}$.

Thus $U_{k + 2} = [u_{1} - \sqrt{(u_{1} - v_{k + 1})^{2} + \tau_{k + 2}},v_{k + 1}]$. Then we may choose $u_{k + 2} \in U_{k + 2}$ such that

$$ u_{k + 2} = \frac{u_{1} + v_{k + 1} - \sqrt{(u_{1} - v_{k + 1})^{2} + \tau_{k + 2}}}{2}. $$

Now, we show that $v_{k + 2} > 0$.

Since

$$\begin{aligned} v_{k + 2} &= \frac{u_{k + 2} + \varepsilon_{k + 2}}{1 +2s_{k + 2}} \\ &= \frac{\frac{u_{1} + v_{k + 1} - \sqrt{(u_{1} - v_{k + 1})^{2} +\tau_{k + 2}}}{2} +\frac{1}{k + 2}}{1+2^{k+2}} \\ &= \frac{1}{(k + 2)(1 + 2^{k + 2})} + \frac{1 + v_{k + 1} - \sqrt{(u _{1} - v_{k + 1})^{2} + \frac{1}{k + 2}}}{2(1 + 2^{k + 2})}, \end{aligned}$$

then

$$\begin{aligned} v_{k + 2} > 0 &\quad \Leftrightarrow \quad \frac{1}{k + 2} + \frac{1 + v_{k + 1}}{2} > \frac{\sqrt{(1 - v_{k + 1})^{2} + \frac{1}{k + 2}}}{2} \\ &\quad \Leftrightarrow \quad \frac{1}{(k + 2)^{2}} + \frac{1}{k + 2} + \frac{v_{k + 1}}{k + 2} + v_{k + 1} > \frac{1}{4(k + 2)}, \end{aligned}$$

which is obviously true. Thus $v_{k + 2} > 0$.

Next, we show that $v_{k + 1} > \frac{1}{2^{k + 2}(k + 2)}$.

Since $v_{k + 1} = \frac{u_{k + 1} + \varepsilon_{k + 1}}{1 + 2s_{k + 1}} = \frac{(k + 1)u_{k + 1}}{(k + 1)(1 + 2^{k + 1})} + \frac{1}{(k + 1)(1 + 2^{k + 1})}$, then

2.13 $$\begin{aligned} \begin{aligned}[b] &v_{k + 1} > \frac{1}{2^{k + 2}(k + 2)} \\ &\quad \Leftrightarrow\quad (k + 1)u_{k + 1} + 1 > \frac{(k + 1)(1 + 2^{k + 1})}{2^{k + 2}(k + 2)} \\ &\quad \Leftrightarrow\quad (k + 1)\frac{1 + v_{k} - \sqrt{(1 - v_{k})^{2} + \frac{1}{k + 1}}}{2} > \frac{k + 1 - k2^{k + 1} - 3\cdot 2^{k + 1}}{2^{k + 2}(k + 2)} \\ &\quad \Leftrightarrow\quad (1 + v_{k}) + \frac{3 + k}{(k + 1)(k + 2)} - \frac{1}{2^{k + 1}(k + 2)} > \sqrt{(1 - v_{k})^{2} + \frac{1}{k + 1}} \\ &\quad \Leftrightarrow \quad \biggl[\frac{3 + k}{(k + 1)(k + 2)} - \frac{1}{2^{k + 1}(k + 2)} \biggr]^{2} + 4v_{k} + 2v_{k}\biggl[ \frac{3 + k}{(k + 1)(k + 2)} - \frac{1}{2^{k + 1}(k + 2)}\biggr] \\ &\qquad \quad \quad {}+ 2\biggl[\frac{3 + k}{(k + 1)(k + 2)} - \frac{1}{2^{k + 1}(k + 2)}\biggr] > \frac{1}{k}. \end{aligned} \end{aligned}$$

Note that

$$ 2 \biggl[\frac{3 + k}{(k + 1)(k + 2)} - \frac{1}{2^{k + 1}(k + 2)} \biggr] - \frac{1}{k+1} = \frac{k2^{k + 1} + 2^{k + 3} - 2k - 2}{2^{k + 1}(k + 1)(k + 2)} > 0, $$

then (2.13) is true, which implies that $v_{k + 1} > \frac{1}{2^{k + 2}(k + 2)}$.

Finally, we show that $v_{k + 2} < v_{k + 1}$.

From the definition of $u_{k + 2}$, we have $u_{k + 2} < \frac{1 + v _{k + 1} - (1 - v_{k + 1})}{2} = v_{k + 1}$. Then $v_{k + 2} < \frac{v _{k + 1} + \frac{1}{k + 2}}{1 + 2^{k + 2}}$. Since $v_{k + 1} > \frac{1}{2^{k + 2}(k + 2)}$, then $\frac{v_{k + 1} + \frac{1}{k + 2}}{1 + 2^{k + 2}} - v_{k + 1} = \frac{\frac{1}{k + 2} - 2^{k + 2}v_{k + 1}}{1 + 2^{k + 2}} < 0$, which implies that $v_{k + 2} < v_{k + 1}$.

Therefore, by induction, (2.12) is true for $n \in N$. Since $0 < v_{n + 1} < v_{n} < 1$, then $\lim_{n \to \infty } v_{n}$ exists. Set $a = \lim_{n \to \infty } v_{n}$. From (2.12), $\lim_{n \to \infty } u_{n} = a$ and from (2.6), $a = 0$. Then in view of (2.7), $\lim_{n \to \infty } w_{n} = 0$. That is, $\lim_{n \to \infty } w_{n} = \lim_{n\to\infty } v_{n} = \lim_{n \to \infty } u_{n} = 0$.

This completes the proof. □

#### Remark 2.16

**Figure 1:**
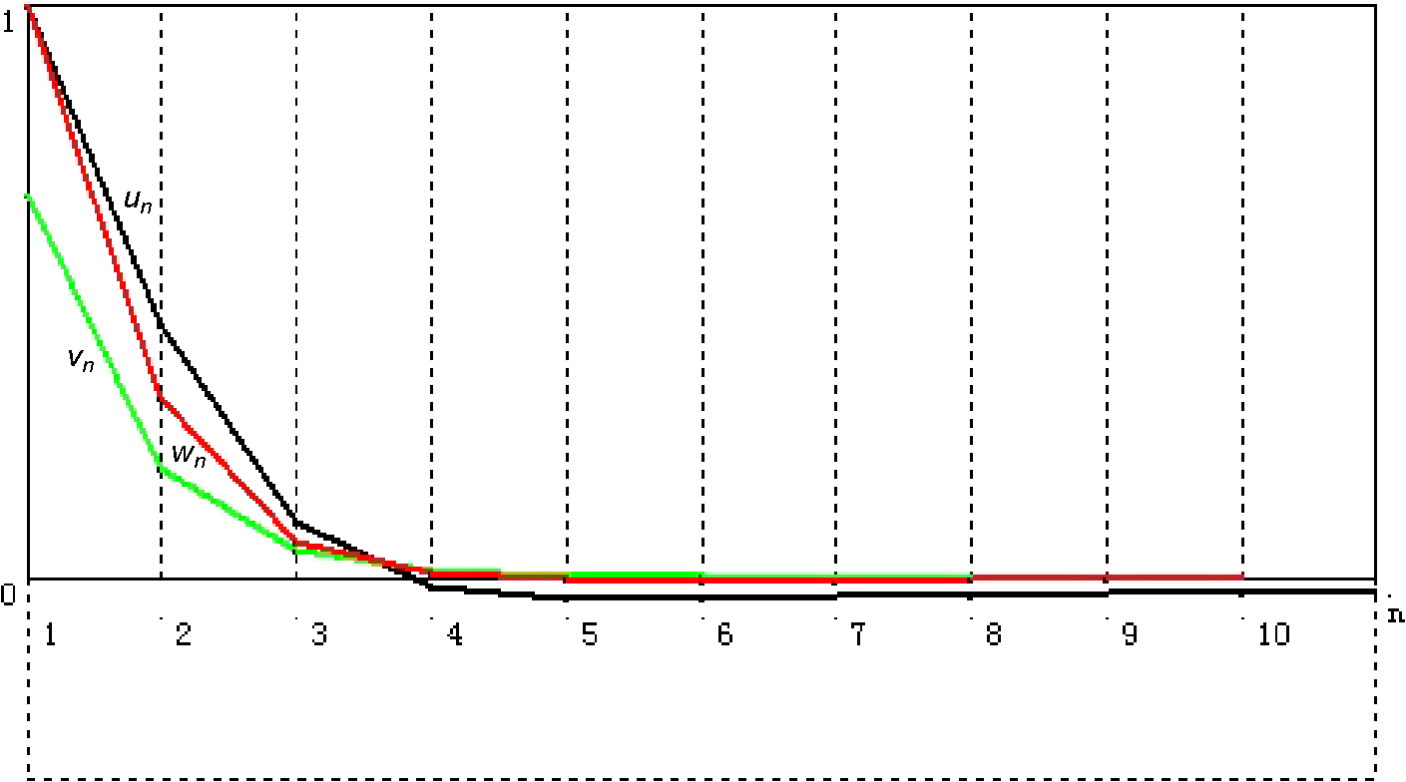
Convergence of $\{ u_{n}\}$, $\{ v_{n}\}$, and $\{ w_{n} \}$

We next do a computational experiment on (2.5) in Remark 2.15 to check the effectiveness of iterative algorithm (2.3). By using the codes of Visual Basic Six, we get Table 1 and Fig. 1, from which we can see the convergence of $\{ u_{n}\}$, $\{ v_{n}\}$, and $\{ w_{n}\}$.

**Table 1:** Numerical results of $\{ u_{n}\}$, $\{ v_{n}\}$, and $\{ w_{n}\}$ with initial $u_{1} = 1.0$

*n*	$v_{n}$	$w_{n}$	$u_{n}$
1	0.666666666666667	1.00000000000000	1.00000000000000
2	0.188493070669609	0.315479212008828	0.442465353348047
3	0.047734978022387	0.063917141637640	0.096281468868147
4	0.013887781581545	0.006938907907725	−0.01390771311373
5	0.005016751133393	−0.00287604161289	−0.03444721259803
6	0.002022073632571	−0.00418691873111	−0.03523188054954
7	0.000854971429905	−0.00391942854572	−0.03256582839944
8	0.000371596957448	−0.00362300404227	−0.02949958193595
9	0.000164574841194	−0.00281862431655	−0.02668421757849
10	0.000073908605586	−0.002357850182411	−0.02424367927438

#### Remark 2.17

Similar to Remark 2.15, considering the same example in Remark 2.14 and choosing the initial value $u_{1} = 1 \in ( - \infty, + \infty )$, we can get an iterative sequence $\{ u_{n}\}$ by algorithm (2.4) in the following way:

2.14 $$\begin{aligned} \textstyle\begin{cases} u_{1} = 1 \in ( - \infty, + \infty ), \\ u_{n + 1} = \frac{u_{1} + v_{n} - \sqrt{(u_{1} - v_{n})^{2} + \tau_{n + 1}}}{2},\quad n \in N, \end{cases}\displaystyle \end{aligned}$$

where $v_{n} = \frac{u_{n} + \varepsilon_{n}}{1 + 2s_{n}}$ and $w_{n} = \alpha_{n}u_{1} + (1 - \alpha_{n})v_{n}$ for $n \in N$. Then $\{ u_{n}\}$, $\{ v_{n}\}$, and $\{ w_{n}\}$ converge strongly to $0 \in N(T) \cap F(S)$, as $n \to \infty $.

#### Remark 2.18

**Figure 2:**
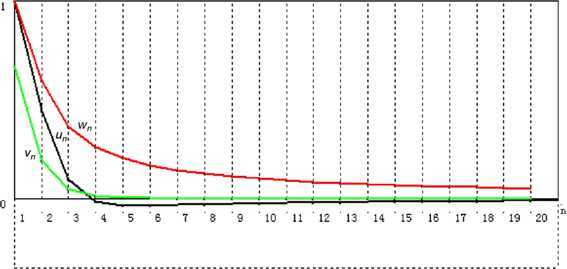
Convergence of $\{ u_{n}\}$, $\{ v_{n}\}$, and $\{ w_{n} \}$

We do a computational experiment on (2.14) in Remark 2.17 to check the effectiveness of iterative algorithm (2.4). By using the codes of Visual Basic Six, we get Table 2 and Fig. 2, from which we can see the convergence of $\{ u_{n}\}$, $\{ v_{n}\}$, and $\{ w_{n}\}$.

**Table 2:** Numerical esults of $\{ u_{n}\}$, $\{ v_{n}\}$, and $\{ w_{n}\}$ with initial $u_{1} = 1.0$

*n*	$v_{n}$	$w_{n}$	$u_{n}$
1	0.666666666666667	1.00000000000000	1.00000000000000
2	0.188493070669609	0.594246535334805	0.442465353348047
3	0.047734978022387	0.365156652014924	0.096281468868147
4	0.013887781581545	0.260415836186159	−0.01390771311373
5	0.005016751133393	0.204013400906715	−0.03444721259803
6	0.002022073632571	0.168351728027143	−0.03523188054954
7	0.000854971429905	0.143589975511347	−0.03256582839944
8	0.000371596957448	0.125325147337767	−0.02949958193595
9	0.000164574841194	0.111257399858839	−0.02668421757849
10	0.000073908605586	0.100066517745027	−0.02424367927438
11	0.000033552200238	0.090939592909307	−0.02216063262202
12	0.000015364834636	0.083347417765083	−0.02038360583157
13	0.000007086981657	0.076929618752290	−0.01885943628695
14	0.000003288762206	0.071431625279192	−0.01754220267938
15	0.000001534136645	0.066668098527535	−0.01639454294823
16	0.000000718881060	0.062500673950994	−0.01538669196834
17	0.000000338196904	0.058823847714733	−0.01449504667360
18	0.000000159662486	0.055555706347903	−0.01370083322728
19	0.000000075612039	0.052631650579827	−0.01298901840146
20	0.000000035908223	0.050000034112812	−0.01234746359706

### Applications to minimization problems

Let $h:E \to ( - \infty, + \infty]$ be a proper convex, lower-semicontinuous function. The subdifferential *∂h* of *h* is defined as follows: $\forall x \in E$,

$$ \partial h(x) = \bigl\{ z \in E^{*}:h(x) + \langle y - x,z \rangle \le h(y), \forall y \in E \bigr\} . $$

#### Theorem 2.19

*Let* *E*, *S*, $\{ \varepsilon_{n}\}$, $\{ s_{n}\}$, $\{ \tau_{n}\}$, *and* $\{ \alpha_{n}\}$ *be the same as those in Corollary *2.3. *Let* $h:E \to ( - \infty, + \infty]$ *be a proper convex*, *lower*-*semicontinuous function*. *Let* $\{ u_{n}\}$ *be generated by*

$$ \textstyle\begin{cases} u_{1} \in E,\quad \varepsilon _{1} \in E, \\ v_{n} = \arg \min_{z \in E}\{ h(z) + \frac{1}{2s_{n}}\Vert z \Vert ^{2} - \frac{1}{s_{n}} \langle z,J(u_{n} + \varepsilon_{n}) \rangle \}, \\ w_{n} = J^{ - 1}[\alpha_{n}Ju_{n} + (1 - \alpha_{n})JSv_{n}], \\ V_{1} = W_{1} = E, \\ V_{n + 1} = \{ z \in E: \langle v_{n} - z,J(u_{n} + \varepsilon _{n}) - Jv_{n} \rangle \ge 0\} \cap V_{n}, \\ W_{n + 1} = \{ z \in V_{n + 1}:\varphi (z,w_{n}) \le \alpha_{n}\varphi (z,u _{n}) + (1 - \alpha_{n})\varphi (z,v_{n})\} \cap W_{n}, \\ U_{n + 1} = \{ z \in W_{n + 1}:\Vert u_{1} - z \Vert ^{2} \le \Vert P_{W_{n + 1}}(u_{1}) - u_{1} \Vert ^{2} + \tau_{n + 1}\}, \\ u_{n + 1} \in U_{n + 1},\quad n \in N. \end{cases} $$

*Then*

1. *if* $v_{n} = u_{n} + \varepsilon_{n}$ *and* $w_{n} = J^{ - 1}[\alpha_{n}Ju_{n} + (1 - \alpha_{n})J(u_{n} + \varepsilon_{n})]$ *for all* $n \in N$, *then* $u_{n} + \varepsilon_{n} \in N(\partial h) \cap F(S)$.
2. *If* $N(\partial h) \cap F(S) \ne \emptyset $ *and* $\inf_{n}s _{n} > 0$, *then the iterative sequence* $u_{n} \to y_{o} = P_{\bigcap_{n = 1}^{\infty }W_{n}} (u_{1}) \in N(\partial h) \cap F(S)$, *as* $n \to \infty $.

#### Proof

Similar to [11], $v_{n} = \arg \min_{z \in E}\{ h(z) + \frac{1}{2s_{n}}\Vert z \Vert ^{2} - \frac{1}{s_{n}} \langle z,J(u _{n} + \varepsilon_{n}) \rangle \}$ is equivalent to $0 \in \partial h(v_{n}) + \frac{1}{s_{n}}Ju_{n} - \frac{1}{s_{n}}J(u_{n} + \varepsilon_{n})$. Then $v_{n} = (J + s_{n}\partial h)^{ - 1}J(u_{n} + \varepsilon_{n})$. So, Corollary 2.3 ensures the desired results.

This completes the proof. □

#### Theorem 2.20

*We only do the following changes in Theorem *2.19: $w_{n} = J^{ - 1}[\alpha_{n}Ju_{1} + (1 - \alpha_{n})JSv_{n}]$ *and* $W_{n + 1} = \{ z \in V_{n + 1}:\varphi (z,w_{n}) \le \alpha_{n}\varphi (z,u_{1}) + (1 - \alpha_{n})\varphi (z,v_{n})\} \cap W_{n}$. *Then*, *under the assumptions of Corollary *2.6, *we still have the result of Theorem *2.19.
